# A Retrospective Toxicology Study of Polysubstance Use Patterns Associated with Xylazine

**DOI:** 10.3390/ijms27041822

**Published:** 2026-02-14

**Authors:** Wanzhu Zhao, Carlos Goncalves, Emily Ruggiano, Trenton Deanna, Elnaz Navid, Fabiola Estrada, Austin Rawlings, Monte Thompson, Andrew Monte, Uwe Christians

**Affiliations:** 1 iC42 Toxicology, Department of Anesthesiology, School of Medicine, University of Colorado Anschutz Medical Campus, Aurora, CO 80045, USA; 2 Rocky Mountain Poison & Drug Safety, Denver, CO 80045, USA

**Keywords:** LC-MS/MS, xylazine, polysubstance abuse, biomarker, urine drug testing, novel psychoactive substances

## Abstract

In recent years, xylazine has emerged as a cutting agent combined with illicit drugs to extend their effects. The present study aimed to discover drug use patterns associated with xylazine-positive and -negative urine toxicology drug screens and to assess whether xylazine can be used as a marker for exposure to designer drugs/new psychoactive substances in our study population. This is a retrospective analysis of urine toxicology results from two different analytical platforms: a targeted, structurally confirmatory, high-performance liquid chromatography–tandem mass spectrometry (LC-MS/MS) assay that quantifies 136 drugs and metabolites including xylazine; and a non-targeted ThermoFisher Orbitrap Tribrid mass spectrometry system (Thermo Scientific^TM^, Bremen, Germany) in combination with database searches for the identification of drugs not captured by the targeted assay. All participants were patients receiving care through the Addiction Research and Treatment Services (ARTS), with documented substance misuse, undergoing routine urine drug toxicology testing at the iC42 Clinical Toxicology. Data analysis was performed using Sciex OS version 2.2.0.5738 after extraction using the targeted, structurally confirmatory and quantitative LC-MS/MS platform (SCIEX, Framingham, MA, USA). The drug patterns found in xylazine-positive and -negative urine samples were statistically significantly different (*p* < 0.001), indicating different consumption patterns associated with xylazine. Moreover, the overall concentrations of drugs (normalized to creatinine) were also statistically significantly different with higher concentrations in the urine samples that tested negative for xylazine. In contrast, samples that were positive for xylazine contained significantly higher concentrations of various designer drugs/new psychoactive substances as detected by the untargeted platform (*p* < 0.0001). The results indicated that xylazine has become increasingly common in Denver’s drug circulation and that xylazine may be used as a marker to prompt reflex testing with non-targeted high-resolution mass spectrometry assays in combination with database searches to test for the exposure to designer drugs/new psychoactive substances in our patient population.

## 1. Introduction

**Figure 1:**
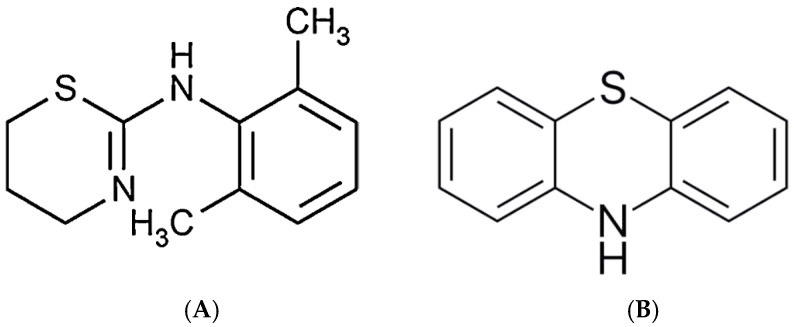
(**A**) Xylazine structure. (**B**) Phenothiazine.

Xylazine [1] was synthesized first in Germany in 1962 as a potential pharmacological treatment for human hypertension; however, it was quickly rejected in the clinical setting due to its association with profound respiratory depression, central nervous system depression, bradycardia, decreased cardiac output, and even death [2]. Since then, xylazine has been exclusively used in veterinary medicine as a tranquilizer, especially for large animals prior to surgical procedures. Xylazine stimulates α2-receptors in the central nervous system via a potent agonistic action and, thus, decreases the concentration of norepinephrine and dopamine in the central nervous system [2,3]. The result is marked sedation, muscle relaxation and anesthetic effects [3,4]. Structurally, xylazine is similar to the class of compounds known as phenothiazines [5] (Figure 1), which includes several commonly prescribed anti-psychotic drugs to treat schizophrenia, anxiety and hyperemesis. Xylazine has emerged over the years as an adulterant with increasing popularity that is associated with clinical presentation of skin ulcers, amnesia, hypotension, bradycardia, and increased overdose death when associated with other illicit drugs [6,7,8,9].

Geographically, according to the State Unintentional Drug Overdose Reporting System (SUDORS), xylazine was first identified as a street drug additive in Puerto Rico in the early 2000s [10]. It quickly spread to numerous states across the United States with Philadelphia exhibiting the highest prevalence of xylazine-related deaths (25.8%) [8]. In Colorado, there has also been an increasing clinical representation of xylazine-related skin necrosis. The Colorado Department of Public Health and Environment has recorded xylazine-associated drug overdoses since 2022; there have been four drug overdose deaths in the Denver Metro area, all in combination with fentanyl, methamphetamine, or other drugs in which xylazine was also detected. The role of xylazine in these deaths is unknown [11]. However, there is no program in place to systematically monitor xylazine use and its association with other illicit drugs. To answer this question, we retrospectively analyzed a database of comprehensive urine toxicology screening results that were generated with a validated, targeted, and structurally confirmatory high-performance liquid chromatography–tandem mass spectrometry (LC-MS/MS) assay that quantifies 136 pain drugs and drugs of abuse [12]. Xylazine was incorporated into the said platform and validated following applicable FDA Guidance for Industry and Clinical Laboratory Standards Institute guidelines [13,14]. The validated analytical measuring range for xylazine in human urine was 10–1000 ng/mL. Intra- and inter-day trueness and imprecision met the predefined acceptance criteria recommended by the FDA M10 guideline [13]: intra-day and inter-day trueness were 85–115% of the nominal concentration (at the lower limit of quantification: 80–120%) and intra-day and inter-day imprecision were <15% (at the lower limit of quantification: <20%). There were no significant matrix interferences or carry-over and extracted samples were stable in the autosampler at +4 °C for at least 24 h.

All samples were received from the University of Colorado Department of Psychiatry Addiction Research and Treatment Services (ARTS), which are a network of stationery and mobile addiction treatment clinics across the Denver Metro area. Taken together, the emergence of xylazine as an adulterant in the illicit drug supply, its documented clinical harms, and the limited monitoring infrastructure in Colorado underscore the need for systematic data collection and subsequent public health responses. By leveraging comprehensive toxicology screening data from a large addiction treatment network, this study aimed to characterize the prevalence of xylazine use and its co-occurrence with other substances. These findings can inform evidence-based harm reduction strategies and guide policymakers, clinicians, and community programs in mitigating the risks associated with this evolving public health threat.

## 2. Results

**Figure 2:**
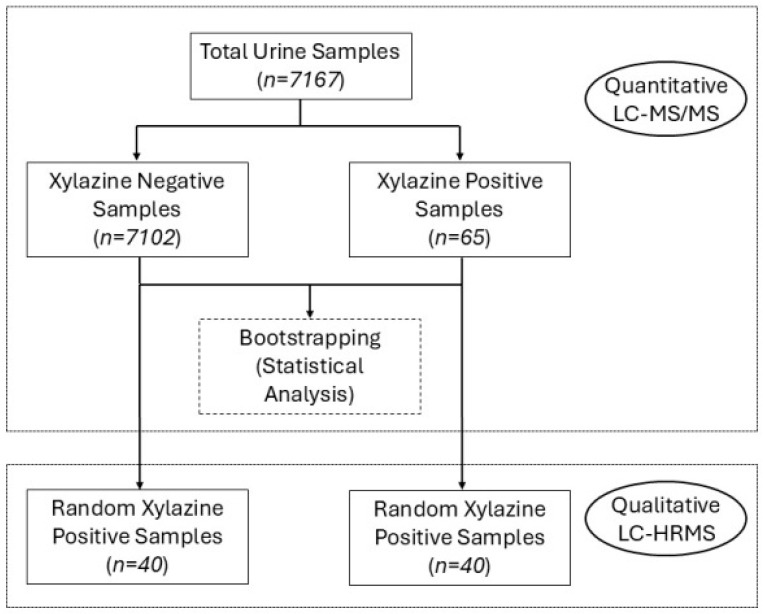
Overview of the selection and analytical workflow used to evaluate xylazine-positive and xylazine-negative urine samples through statistical bootstrapping and a combination of targeted, quantitative and confirmatory LC-MS/MS and LC-HRMS (qualitative).

In the quantitative analysis, 7167 unique urine samples were analyzed, of which 65 tested positive for xylazine. The retrieved dataset included 65 samples from 50 different individuals that had urine samples positive for xylazine; 7102 datasets from 1752 individuals were retrieved whose urine samples had tested negative for xylazine. Another random set of 40 samples that were negative for xylazine were also analyzed using a non-targeted high-resolution MS/MS assay (*vide infra*). Figure 2 presents the experimental design with the number of samples analyzed using the quantitative LC-MS/MS and the non-targeted, screening LC-high-resolution mass spectrometry assay (LC-HRMS).

The drug patterns found in xylazine-positive and -negative urine samples were statistically significantly different indicating different use patterns. A total of 61 different drugs in addition to xylazine were detected in the urine samples collected from those patients who tested positive for xylazine; the total number of different drugs in the xylazine-negative samples was 105 (*p* < 0.001). Moreover, in addition to different use patterns, the overall concentrations of drugs as determined using the quantitative LC-MS/MS assay (normalized to creatinine) were also statistically significantly different with higher concentrations in the urine samples that tested negative for xylazine. In contrast, samples that were positive for xylazine contained significantly higher concentrations of various designer drugs/new psychoactive substances (*p* < 0.0001).

**Figure 3:**
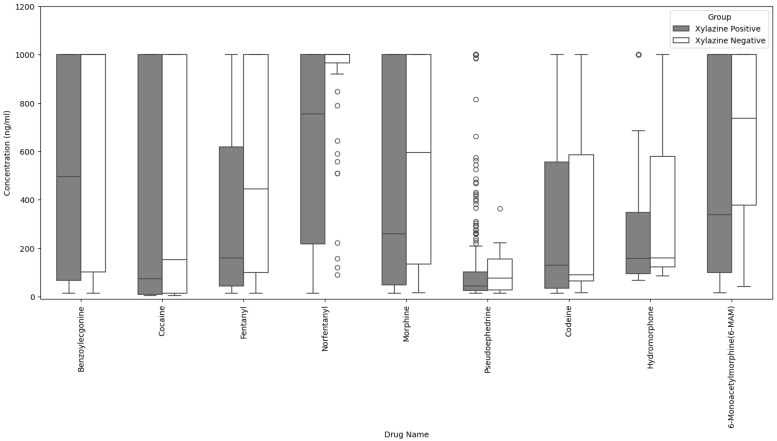
Concentrations of selected drugs (and key metabolites if applicable) between xylazine-positive vs. -negative samples. One thousand (1000 ng/mL) was the upper limit of quantification of the quantitative LC-MS/MS assay [10] and, accordingly, several bars are cut off if concentrations exceeded the upper limit of quantification. Outliers (circles) in this plot represent individual samples with drug concentrations that are substantially higher or lower than the typical range observed within their respective xylazine-positive or xylazine-negative groups.

Xylazine-positive samples exhibited lower concentrations of several co-detected substances (and key metabolites if applicable) compared to xylazine-negative samples, as shown in the boxplots of selected drugs in Figure 3, with median concentrations and interquartile ranges summarized in Table 1.

**Figure 4:**
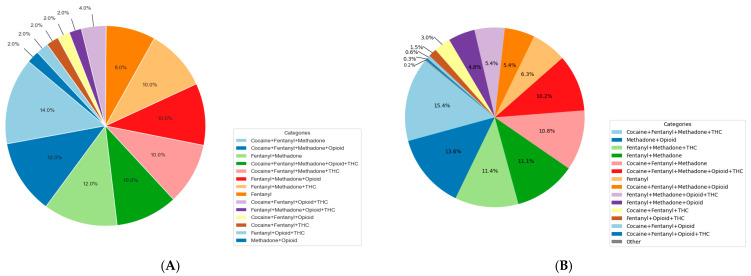
(**A**) Polysubstance use pattern for xylazine-positive urine samples. (**B**) Polysubstance use pattern for xylazine-negative urine samples. Opioid denotes the presence of one or more of the following compounds: codeine, morphine, hydromorphone, or hydrocodone. This graph is based on the results of the targeted, quantitative and structurally confirmatory LC-MS/MS assay measuring 136 drugs and metabolites. Pie chart represents the most frequently identified drugs only.

The xylazine-negative samples exhibited a greater diversity of drug combinations, defined by a larger number of distinct multi-drug patterns and a broader range of co-occurring substances per sample, most frequently involving fentanyl, methadone, cocaine, and THC (Figure 4).

**Figure 5:**
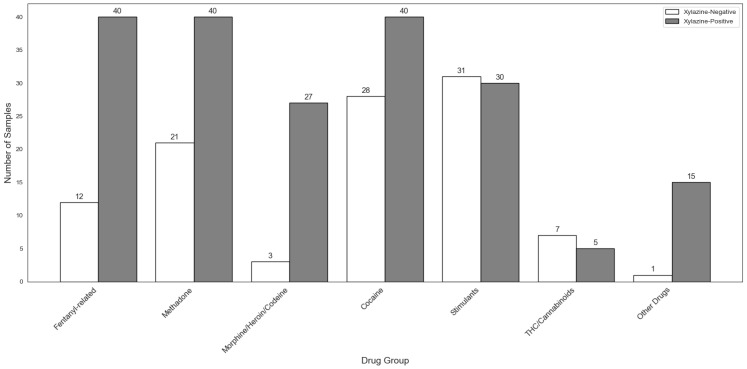
Polysubstance use pattern between xylazine-positive and -negative urine samples including designer drugs/NPSs as measured using the untargeted LC-HRMS assay. Fentanyl-related substances (fentanyl, 4-ANPP, benzyl fentanyl, 3-methylthiofentanyl, beta-hydroxy thio fentanyl, para-fentanyl, etonitazene, meta-methoxy furanyl fentanyl, thio-fentanyl, carfentanil); methadone (methadone, EDDP); morphine/heroin/codeine (morphine, codeine, dihydrocodeine, nor-codeine, 6-mono-acetyl morphine [6-MAM]); cocaine (cocaine, benzoylecgonine); stimulants (methamphetamine, amphetamine, pseudoephedrine, methylphenidate); THC/cannabinoids (THC-COOH, THC-COOH-glucuronide); other drugs (pentazocine, butorphanol tartrate, O-desmethyl tramadol, dextrometorphan, ethyl sulfate, ethyl glucuronide, mitragynine, psilocin).

The fact that xylazine-positive urine samples contained fewer illicit drugs, with a narrower distribution and lower concentrations, compared to xylazine-negative samples led us to hypothesize that drugs were likely being missed in the xylazine-positive samples when using the targeted, quantitative LC–MS/MS assay. Therefore, these samples were further analyzed using the non-targeted LC-HRMS assay in combination with database searches. Indeed, clear differences in drug use patterns were observed between xylazine-positive and xylazine-negative samples (Figure 5). Notably, fentanyl and its analogs were disproportionately represented in the xylazine-positive urine samples, underscoring the strong association between xylazine and high-potency synthetic opioids. In addition, concurrent detection of methamphetamine, benzodiazepines, and cocaine was more common in the xylazine-positive compared to xylazine-negative samples, further illustrating a high-risk polysubstance use pattern.

## 3. Discussion

According to a targeted, validated, and structurally confirmatory LC-MS/MS assay that quantifies 136 drugs and key metabolites, in our study population, xylazine-negative samples exhibited a greater diversity of drug combinations, most frequently involving fentanyl, methadone, cocaine, and THC. Xylazine-positive samples contained lower concentrations of the drugs included in the said assay compared to xylazine-negative samples if the aforementioned assay was used. These findings might indicate that xylazine-positive drugs have a higher psychoactive effect and/or users are trying to evade detection using lower drug doses overall. In this context, xylazine may function as a cutting agent, enhancing or prolonging the psychoactive effects of primary drugs while allowing for lower quantities of those substances, and is associated with a distinct pattern of co-detected new psychoactive substances across multiple drug classes. The use of cutting agents is common in the illicit drug supply chain and is often unknown to users. Before xylazine, lidocaine was frequently used, and more recently, there has been an emerging trend involving medetomidine [15,16,17].

These findings also highlight broader challenges in harm reduction and illicit drug monitoring. Immunoassays are prone to false positives due to cross-reactivities with the antibodies, but also to false negative results due to their limited sensitivity and the fact that immunoassays can only detect drugs if the corresponding antibody is included [18,19,20]. Most current clinical urine toxicology drug monitoring strategies rely on immunoassays combined with LC-MS/MS confirmation. Structurally confirmatory quantitative LC-MS/MS assays are considered the gold standard [12]. Our study used a two-stage analytical approach. First, we used a targeted, structurally confirmatory LC-MS/MS assay that quantifies 136 drugs including xylazine. The limitation of a targeted LC-MS/MS assay is that it is designed to detect only drugs, the ion transitions of which are included in the method (for a list of drugs and metabolites included please see [12]). Moreover, those targeted LC-MS/MS assays are limited by the availability of authentic reference materials. All other drugs are missed (false negatives). We then re-analyzed samples using an LC-HRMS assay. This non-targeted screening assay is highly specific due to the high resolution of the orbitrap detector of 250,000 FWHM (Full Width at Half-Maximum). The number of drugs the LC-HRMS assay was able to detect was only limited by the entries in the database to which the HRMS-spectra were matched. HRMS analysis was combined with the MZCloud database (Watford, UK), which has thousands of entries for drugs and drugs of abuse including designer drugs/NPSs and is constantly updated. The downside is that the LC-HRMS assay is not quantitative and can only confirm the presence or absence of a specific drug. Overall, both assays are complementary and the LC-HRMS assay substantially reduces the risk of missing important drugs. Analysis using the non-targeted LC-HRMS assay further highlighted distinct drug use patterns in xylazine-positive samples in our study population. Concurrent detection of methamphetamine, benzodiazepines, and cocaine was also more common in xylazine-positive than -negative samples, illustrating a high-risk pattern of polysubstance use in patients whose urine tested positive for xylazine.

The emergence of novel psychoactive substances (NPSs), including synthetic/semi-synthetic cannabinoids, nitazene opioids, and other potent analogs, exacerbates the above-mentioned challenges. Some nitazenes, such as etonitazene, are hundreds to thousands of times more potent than morphine or fentanyl. They are frequently added as adulterants to more readily available drugs such as cocaine, heroin, and fentanyl, increasing the risk of overdose. These characteristics underscore the need for easily detectable markers, like xylazine, to identify high-potency drug co-use.

The results of the present study demonstrates that, in our study population, xylazine is associated with polysubstance use and adulteration, particularly with the use of fentanyl, nitazenes, and other synthetic opioids. Monitoring its presence can inform clinical interventions, overdose prevention efforts, and public health strategies.

In a brief report, Hurt et al. reported similar findings in a retrospective analysis [21]. Xylazine was found in 413 out of 59,498 samples that originated from 25 states. This means that 0.7% of the urine samples included in this study tested positive for xylazine, while this was the case in 0.9% of the urine samples included in our study. The substances detected most frequently with xylazine were fentanyl, buprenorphine, naloxone, cocaine, D-methamphetamine, and delta-9-tetrahydrocannabinol and, thus, the pattern differed from that in our study. The designer drugs most frequently discovered by Hurt et al. were fentanyl analogs, isotonitazene, and designer benzodiazepines [21]. Due to the nature of the brief report, not much detail regarding the analytical methodology was reported and, unlike in our study, co-use patterns in xylazine-negative samples were not analyzed [21].

However, the present study has its limitations: the sample is geographically restricted to ARTS clinics in the Denver Metro area, which may overrepresent urban and homeless populations, and the results of the LC-HRMS assay were qualitative and not quantitative. Future studies should incorporate quantitative analyses across broader geographic regions to better understand concentration-dependent effects, the functional role of adulterants like xylazine, and patterns of co-use that drive overdose risk.

## 4. Methods and Materials

### 4.1. Patient Samples

This retrospective database and sample bank analysis included all results from urine toxicology samples received from the University of Colorado Department of Psychiatry Addiction Research and Treatment Services between 24 June 2024 and 5 December 2024. The start date was June 24 since this was the first day that xylazine was routinely tested in urine toxicology screening samples at iC42 Clinical Toxicology (University of Colorado, Aurora, CO, USA). Patients that were undergoing substance use treatment with drug monitoring provided urine samples during their regular visits to monitor for ongoing substance misuse. Samples were transported unrefrigerated by a courier service and were analyzed the same day they were collected during the aforementioned period. Since all datasets were retrospectively retrieved from the results database without any identifying information, the study was considered “exempt” by the Colorado Multi-Institutional Review Board (COMIRB exemption ID: 25-1774). Accordingly, no informed consent was required.

### 4.2. Bioanalytical Assays

Urine samples were collected into cups that were directly placed into a customized Hamilton robotic system (Hamilton, Reno, NV, USA) upon receipt. The robotic system scanned the barcode and fully automatically processed the samples including centrifugation steps to minimize the chance of random errors. A 100 mL aliquot of calibrator, quality control, or patient sample was transferred into a polypropylene 96-deep-well plate. Subsequently, 100 mL of protein precipitation solution was added, and the samples were vortex-mixed for 3 min and centrifuged at 27,500× *g* for 10 min at 4 °C. Following centrifugation, 170 mL of the supernatant was transferred to glass HPLC vials (Phenomenex, Torrance, CA, USA), diluted with 800 mL of HPLC-grade water, briefly vortexed, capped, and placed in the LC–MS/MS autosampler for analysis. The autosampler was maintained at 4 °C, and an 8 µL aliquot was injected for analysis (for more details, please see [12]). Samples were tested for potential adulteration (creatinine, pH, oxidants, specific gravity; all FDA-cleared assays) using an Indiko Plus analyzer (Thermo Fisher, Fremont, CA, USA). Samples that were potentially adulterated (one or more of the following: creatinine < 20 mg/dL; oxidant > 200 µg/dL; pH ∉ [4.5, 8.0]; specific gravity ∉ [1.003, 1.035]) were excluded from the analysis. The routine drug LC-MS/MS analysis was carried out on a Sciex 5500 mass spectrometer (Sciex, Concord, ON, Canda) paired with an Agilent 1290 high-speed binary pump (Agilent Technologies, Santa Clara, CA, USA) and a PAL RSI autosampler (CTC Analytics AG, Zwingen, Switzerland) using a previously validated quantitative LC-MS/MS method [12]. As already mentioned above, xylazine was added to the platform and validated according to the FDA M10 Guidance for the Industry [13] and Clinical Laboratory Standards Institute guidelines [14]. All analyses were carried out in a laboratory that was accredited by the American College of Pathologists (Northfield, IL, USA) and that was CLIA (United States Clinical Laboratory Improvement Amendments)-certified. The assay successfully participated in corresponding proficiency testing programs organized by the American College of Pathologists (www.cap.org, accessed on 1 January 2026) on a regular basis for more than a decade.

After the quantitative analysis was completed on the above-mentioned platform, samples were analyzed again on a ThermoFisher Orbitrap Eclipse Tribrid mass spectrometer equipped with a Vanquish VF-P10-A binary pump as well as a Vanquish VH-C10-A column compartment (all Thermo Fisher, Fremont, CA, USA) after extraction from the previously frozen (−80 °C) archived samples, as described above. The said samples were injected using a Thermo Scientific TriPlus RSI autosampler (CTC Analytics AG, Zwingen, Switzerland) onto a Kinetex 2.6 µm F5 100 mm × 4.6 mm column (Phenomenex, Torrance, CA, USA) using the same high-performance liquid chromatography (HPLC) gradient and mobile phases as used for the aforementioned targeted urine toxicology screening LC-MS/MS assay [12]. As the concentrations of the drugs detected in the targeted LC-MS/MS assay were already known, the LC-HRMS re-analysis was not quantitative and its main purpose was to screen for additional, unknown illicit drugs in the database (*vide infra*) that were not included in the LC-MS/MS assay.

### 4.3. Data Processing and Statistical Methods

Quantitative data analysis was initially carried out using Sciex OS version 2.2.0.5738 (Sciex, Redwood City, CA, USA) after analysis using the targeted, structurally confirmatory and quantitative LC-MS/MS platform. Calibration, quality control and run acceptance criteria followed the rules set forth in the FDA guidance “M10. Bioanalytical Method Validation and Study Sample Analysis” [13]. The quantitative data analysis was performed using a custom programmed and validated laboratory information management system. After data integration and approval of the results, they were stored in a Structured Query Language (SQL) database from which the data for the present analysis was retrieved. The final dataset was then analyzed using Python 3.13.2, and all *p*-values were calculated with the Mann–Whitney test. If a patient had multiple datasets, only the first after admission to the treatment program was used. To address differences in sample size between the xylazine-positive and xylazine-negative groups, a bootstrapping method was applied, randomly sampling the xylazine-negative group to ensure a fair comparison.

The qualitative data analysis used the non-targeted LC-HRMS for the detection of drugs not included in the targeted LC-MS/MS assay including designer drugs and new psychoactive substances. The resulting high-resolution mass spectra were analyzed using ThermoFisher Compound Discovery Software (version 3.4) starting with the comparison of high-resolution MS/MS spectra with an internal iC42 Toxicology compound library that contained the most frequent known designer drugs. Those compounds that did not have a match were further analyzed using the MZCloud library (MZCloud, Watford, UK). Only matches with a score over 90% were selected and preference was given to classes of drugs that were known to be used together with illicit drugs such as fentanyl analogs and nitazenes, as well as other well-known doping agents such as ANPP, MDPV and Khat.

## 5. Conclusions

The present study provides the first systematic evaluation of xylazine in urine toxicology samples from ARTS clinic patients in the Denver Metro area. While the quantitative study showed a lower number of drugs in xylazine-positive compared to xylazine-negative urine samples, the xylazine-positive samples exhibited more complex and diverse drug combinations, most commonly with fentanyl, methadone, cocaine, and THC.

Our findings suggest that xylazine is emerging as a significant adulterant in Colorado’s illicit drug supply, paralleling reports from other regions of the United States [8,9,21,22]. Its frequent co-occurrence with highly potent opioids, including fentanyl analogs and nitazenes, underscores the potential risk of overdose and adverse clinical outcomes in this population. In our study population, the detection of xylazine may serve as a marker for broader polysubstance exposure, offering potential utility in both drug of abuse treatment decision making and overall public health surveillance. Most importantly, our study suggested that, in our clinical practice, the presence of xylazine should trigger reflex testing using a non-targeted LC-HRMS assay in combination with database searches as in our samples the presence of xylazine was associated with a high probability of the presence of designer drugs/NPSs, the knowledge of which may allow for early public health intervention.

## Figures and Tables

**Table 1:** Median analyte concentrations with interquartile ranges in ng/mL.

	Xylazine-Positive Samples	Xylazine-Negative Samples
Fentanyl	160 (44–619)	445 (100–1000)
Morphine	259 (48–1000)	596 (133–1000)
Hydromorphone	157 (94–348)	160 (123–580)
6-Monoacetylmorphine (6-MAM)	338 (100–1000)	736 (378–1000)
Cocaine	75 (9–1000)	152 (13–1000)
Benzoylecgonine	495 (67–1000)	1000 (101–1000)
Codeine	130 (34–555)	91 (65–586)
Norfentanyl	755 (217–1000)	1000 (967–1000)
Pseudoephedrine	43 (26–101)	76 (27–156)

Values are reported as median concentrations with interquartile ranges (IQR); units: ng/mL.

## Data Availability

The original contributions presented in this study are included in the article. Further inquiries can be directed to the corresponding authors.
